# Drug use disorder following early life exposure to tetrachloroethylene (PCE)-contaminated drinking water: a retrospective cohort study

**DOI:** 10.1186/s12940-020-00638-2

**Published:** 2020-09-17

**Authors:** Ann Aschengrau, Alexandra Grippo, Michael R. Winter, Margaret G. Shea, Roberta F. White, Richard Saitz

**Affiliations:** 1 Department of Epidemiology, Boston University School of Public Health, 715 Albany Street, Boston, MA 02118 USA; 2 Biostatistics and Epidemiology Data Analytics Center, Boston University School of Public Health, 85 East Newton Street, Boston, MA 02118 USA; 3 Department of Environmental Health, Boston University School of Public Health, 715 Albany Street, Boston, MA 02118 USA; 4 Department of Neurology, Boston University School of Medicine, 72 East Concord Street, Boston, MA 02118 USA; 5 Department of Community Health Sciences, Boston University School of Public Health, 801 Massachusetts Avenue, Boston, MA 02118 USA; 6 Clinical Addiction Research and Education Unit, Section of General Internal Medicine, Department of Medicine, Boston University School of Medicine, Boston, MA USA; 7 Grayken Center for Addiction, Boston Medical Center, Boston, MA USA

**Keywords:** Tetrachloroethylene, Drinking water, Drug use, Drug use disorder

## Abstract

**Background:**

Many studies of adults with occupational exposure to solvents such as tetrachloroethylene (PCE) have shown adverse effects on cognition, mood and behavioral problems. Much less is known about neurotoxic effects in early life at lower exposure levels seen in community settings. We recently reported that illicit drug use was more frequent among adults from Cape Cod, Massachusetts who were exposed to PCE-contaminated drinking water during gestation and early childhood than their unexposed counterparts. Using newly collected data from this population-based retrospective cohort study, the current analysis examines whether early life PCE exposure is also associated with drug use disorder over the life course.

**Methods:**

Three-hundred and sixty-three subjects with prenatal and early childhood PCE exposure and 255 unexposed subjects were studied. These individuals (median age: 40–41 years) completed self-administered questionnaires on the eleven established diagnostic criteria for drug use disorder and confounding variables. A validated leaching and transport model was used to estimate exposure to PCE-contaminated water.

**Results:**

Overall, 23.3% of subjects reported having at least one criterion for drug use disorder over their lifetime. Early life PCE exposure was associated with a modest increase in the lifetime presence of one or more diagnostic criteria for drug use disorder (adjusted RR: 1.4, 95% CI: 1.0–1.8). Compared to unexposed subjects, PCE-exposed subjects were more likely to report having most diagnostic criteria of drug use disorder, including neglecting major roles due to drug use, physical and psychological problems related to drug use, and giving up activities due to drug use. No dose-response relationships were observed with increasing levels of PCE exposure.

**Conclusions:**

These results suggest that exposure to PCE-contaminated drinking water during early life modestly increases the risk of developing diagnostic criteria for drug use disorder later in life. Because this study has several limitations, these findings should be confirmed in follow-up investigations of other exposed populations with more diverse racial and socioeconomic characteristics.

## Background

From the late 1960’s through the 1980’s, exposure to tetrachloroethylene (perchloroethylene or PCE)-contaminated drinking water was relatively common among New England residents. The contamination resulted from the installation of vinyl-lined asbestos cement (VL/AC) pipes in certain public water distribution systems [1, 2]. The vinyl lining, which was composed of vinyl resin dissolved in PCE (Piccotex; Johns-Manville Corporation, Denver, CO), was introduced to eliminate taste and odor problems in existing AC pipes. Because PCE is volatile, it was expected to evaporate by the time the pipes were installed; however, water samples taken in 1980 revealed that substantial quantities of PCE remained in the liner and were leaching into public drinking water supplies. Although this source of contamination was remediated by flushing and bleeding the affected pipes in the 1980s, other public water supplies across the United States have been contaminated by PCE from landfills and underground storage tanks [3], industrial waste disposal sites [4], and dry cleaning facilities [5].

PCE’s potential to cause neurotoxic effects has been established through numerous animal and human studies [6, 7]. Studies of adults exposed to PCE and related solvents in occupational settings have reported decrements in cognition, memory, attention, executive function, and trigeminal nerve and vestibular function [8–15]. Studies have also linked occupational exposure to increases in anxiety and depression [8, 9, 11, 16–18].

The few studies of cognition and other behavioral effects among individuals with early life exposure to organic solvents have produced mixed results. Eskenazi et al. found no changes in intellectual ability or memory among children with prenatal solvent exposure [19]. In addition, no meaningful deficits were seen in studies of cognitive and behavioral function among day care attendees who were exposed to PCE from a nearby dry cleaners [20, 21]. In contrast, other studies found that, compared to unexposed children, those with prenatal solvent exposure had lower scores on language tests, more problem behaviors [22], lower scores on tests of intelligence and language skills [23], and a greater frequency of behaviors indicative of attention deficits, hyperactivity, and aggression [24, 25].

Our own population-based retrospective cohort study (“Cape Cod Health Study”) found long-lasting neurotoxic effects of prenatal and early childhood exposure to PCE contaminated drinking water. In particular, we found increased risks of mood disturbances and diminished performance on tests of learning, memory, attention, and executive functioning [26] and illicit drug use [27] among exposed individuals. Specific drugs for which increased risks were observed included crack/cocaine, psychedelics/hallucinogens, club/designer drugs and heroin. Because using these drugs may lead to further uncontrollable use [28, 29], we used newly collected data to examine the association between early life PCE exposure and the diagnostic criteria for drug use disorder. The hypothesis that an environmental exposure such as PCE could be part of the multifactorial etiology of drug use disorder [30, 31] is novel but supported by experimental evidence that PCE and related solvents could increase the risk of drug use and drug use disorder by altering brain function via peroxidation of cell membrane lipids [32], changes in the fatty acids in the brain [33], demyelination [34], and negative impacts on brain receptors (e.g., [35–39]).

## Methods

### Study population and follow-up

The Cape Cod Health Study was approved by the Institutional Review Boards of the Massachusetts Department of Public Health and Boston University Medical Center. Eligible individuals were born during 1969–1983 to married women living in Cape Cod, Massachusetts towns known to have some VL/AC water pipes in their water distribution system. The details of study selection have been previously described [27]. In brief, two groups of “index” subjects were selected: (1) those born to women who were exposed to PCE-contaminated drinking water at birth (*N* = 1910), and (2) those born to women who were unexposed at their birth (*N* = 1928). These groups were identified by cross-matching maternal addresses on birth certificates with information from local water companies on the location and installation year of the VL/AC pipes. Unexposed subjects were randomly selected and frequency matched to exposed subjects on month and year of birth. In addition, older siblings of exposed and unexposed subjects who were born in Massachusetts during 1969–1983 (*N* = 1202) were identified. These siblings were initially considered unexposed because they were born before the family moved to an affected residence, but final exposure designations for all subjects were assigned after more extensive exposure assessments were conducted, as described below.

Birth certificates of all individuals were reviewed to obtain information on the study family, including the names of the subject and parents; the subject’s date of birth, birth weight and gestational duration; and the parents’ ages and educational levels when the subject was born. During Phase 1 of the study (2000–2005), individuals were traced and sent invitation letters describing the purpose of the study and requesting that they complete a self-administered questionnaire. Overall 40.5% of successfully located subjects returned the study questionnaire (*N* = 1689). The Phase 1 questionnaire gathered information on cigarette smoking, alcoholic beverage consumption, and drug use, demographic and medical characteristics; and occupational and non-occupational exposure to solvents. Information was also collected on the family’s residences from the subject’s birth through 1990, including the exact street address and calendar years of residence for all Cape Cod addresses. Lastly, the survey collected information on the subject’s knowledge of the PCE contamination episode and knowledge of their own PCE exposure.

Phase 2 of the study (2017–2020) focused on the 1512 subjects from Phase 1 who had adequate residential data for assessing their PCE exposure status. A total of 694 subjects completed the entire Phase 2 survey (46.8%). The remainder never responded to several contact attempts (*N* = 718), were not found (*N* = 54), were deceased (*N* = 6), declined to participate (*N* = 26), or completed out only a small portion of the survey (*N* = 14). The Phase 2 questionnaire updated information on cigarette smoking, alcoholic beverage consumption and drug use; and demographic and medical characteristics. Drug information included use of cannabis, cocaine, heroin, hallucinogens, inhalants, and methamphetamine, and misuse of prescription pain relievers, tranquilizers, stimulants and sedatives. The Phase 2 questionnaire also collected new information on the eleven established Diagnostic and Statistical Manual of Mental Disorders Fifth Edition (DSM5) criteria for drug use disorder for any drug. Questions for these criteria, which were adapted from the validated Alcohol Use Disorder and Associated Disabilities Interview Schedule (AUDADIS) [40], ascertained the lifetime prevalence of the following behaviors: ever engaging in hazardous use that increased the chances of getting hurt, ever having social/interpersonal problems due to use, often neglecting major roles due to use, ever developing tolerance such that the usual drug amount had much less of an effect than it once did, often using larger amounts or for a longer period of time than intended, ever making unsuccessful attempts to stop or cut down use, ever spending much time getting or using the drug, ever continuing to use a drug despite having physical and psychological problems related to use, ever giving up activities due to use, ever developing cravings for the drug, and ever having withdrawal symptoms when the drug effects were wearing off. We were unable to obtain physician confirmation of these criteria.

Comparison of Phase 2 participants (*N* = 694) and non-participants (*N* = 818) found that they were quite similar with regard to many characteristics, including PCE exposure status, age, race, maternal age, birth weight, gestational age, and receipt of prenatal care (Table 1). However, compared to Phase 2 non-participants, a larger proportion of Phase 2 participants were female, had mothers who were college graduates and fathers employed in white collar jobs, and a smaller proportion smoked cigarettes regularly, reported heavy episodic drinking and used drugs including crack/cocaine, heroin, psychedelics/hallucinogens, club/designer drugs, inhalants, and Ritalin without a prescription (Table 1).

**Table 1:** Distribution of Selected Characteristics by Phase 2 Participation Status

Characteristic	Participant (N = 694)	Non-Participant (N = 818)
PCE exposure status		
Both pre and postnatal exposure	363 (52.3%)	468 (57.2%)
Only postnatal exposure	76 (11.0%)	58 (7.1%)
Unexposed	255 (36.7%)	292 (35.7%)
Age (Phase 2, mean, sd)	41 (4)	40 (4)
White race	688 (99.1%)	802 (98.0%)
Sex		
Male	242 (34.9%)	360 (44.0%)
Female	452 (65.1%)	458 (56.0%)
Ever smoked on a regular basis (Phase 1 data)	202 (29.4%)	343 (42.3%)
Missing	6	8
Drank 5 + (men)/4 + (women) drinks^*^ per day, past 30 days (Phase 1 data)	80 (11.8%)	147 (18.7%)
Missing	16	32
Any drug use** (Phase 1 data)	230 (33.1%)	351 (42.9%)
Mother’s age at subject’s birth (Mean, SD)	27 (4)	27 (5)
Mother’s educational level at subject’s birth		
High school graduate or less	230 (33.2%)	340 (41.7%)
Some college	210 (30.3%)	254 (31.1%)
4 year college graduate or higher	253 (36.5%)	222 (27.2%)
Missing	1	2
Father’s occupation at subject’s birth		
White collar	371 (53.9%)	370 (46.0%)
Blue collar	211 (30.7%)	285 (35.4%)
Other	106 (15.4%)	149 (18.5%)
Missing	6	14
Subject’s birth weight (Mean, SD)	3423 (524)	3438 (507)
Missing	60	44
Subject’s gestational age (weeks) (Mean, SD)	40 (2)	40 (2)
Missing	29	60
Mother received prenatal care during subject’s pregnancy	663 (99.8%)	764 (99.6%)
Missing	30	51

^*^A drink was defined as 12-oz bottle, can, or glass of beer, 4-oz glass of wine, 12-oz bottle of wine cooler, hard lemonade, or hard cider, shot of liquor straight or in a mixed drink

**Included crack/cocaine, heroin, psychedelics/hallucinogens, club/designer drugs, inhalants, and Ritalin without a prescription

### PCE exposure assessment

PCE exposure assessments were conducted during Phase 1. As a first step, all residential addresses on Cape Cod reported by subjects were geocoded to a latitude and longitude using ArcGIS 8.1. Approximately 95% of reported addresses were successfully geocoded without knowledge of the exposure or outcome status. Visual inspection of the water distribution maps determined initial exposure status and was followed by a more detailed assessment using a leaching and transport model developed by Webler and Brown [41]. The model estimates the amount of PCE entering a residence by using the initial amount of PCE in the liner, the age of the pipe, and the leaching rate of PCE from the liner into the water. This rate is modeled as an exponential relationship with a rate constant of 2.25 years based on experimental data [42]. Additional model parameters include the water flow rate and direction which were determined using EPANET, water distribution system modeling software developed by the U.S. EPA. This software has been used in other epidemiological studies of drinking water contaminants [43, 44]. We were able to calculate only annual PCE exposures because only move-in and pipe installation years were available. We estimated PCE exposure during the prenatal period by multiplying the annual mass of PCE that entered the subject’s residence during their birth year by 9/12. We estimated cumulative exposure during early childhood by summing the estimated mass of PCE that entered their residences from the month and year following birth through the month and year of the fifth birthday. Simple percentages were used to account for partial years.

### Statistical analysis

We compared the criteria for drug use disorder among subjects with prenatal and early childhood exposure combined (*N* = 363) to unexposed subjects (*N* = 255). Nearly all subjects with prenatal exposure also had childhood exposure and so we were unable to examine the impact of prenatal exposure alone. In addition, the number of subjects exposed only during childhood was too small to provide stable results (*N* = 76). In addition, we examined early life PCE exposure in relation to the lifetime presence of any criteria (> = 1), two or more criteria, and finer groupings of the number of criteria (1, 2–3, 4–5, and 6 or more) overall and stratified by sex. The lifetime presence of two or more criteria was examined because the formal diagnosis of a drug use disorder requires the presence of two or more criteria, albeit in a single year. We also examined specific criteria (e.g., neglected major roles due to use, physical and psychological problems related to use) and the impact of PCE exposure levels (> = median and > 0- < median) to determine if a dose-response relationship was present. Lastly, we compared the drug use history among individuals with and without any criteria for drug use disorder.

The risk ratio (RR) was used to estimate the strength of the association between PCE exposure and the criteria. Ninety-five percent confidence intervals were used to assess their precision. First, crude analyses were conducted and then generalized estimating equation (GEE) analyses were performed to account for non-independent outcomes arising from several children from the same family [45, 46]. Twelve percent of Phase 2 subjects were siblings. Lastly, adjusted GEE analyses were conducted to control for confounding variables. Covariates considered for these analyses were factors associated with drug use disorder that, according to our directed acyclic graph (DAG), also influenced their PCE exposure. While many variables were risk factors for having criteria for drug use disorder, only those with plausible causal relationships with PCE exposure were ultimately controlled. These variables, which reflected calendar time and socioeconomic status, were the subject’s age, mother’s educational level and father’s occupation.

## Results

As shown in Table 2, the characteristics of exposed and unexposed subjects were quite similar. Subjects were, on average, 40–41 years old when they completed the Phase 2 questionnaire, and were mainly female, white, college-educated, married or cohabitating, and employed. Few subjects had possible occupational exposure to solvents while many had potential solvent exposure from hobbies. Comparable proportions of exposed and unexposed subjects reported a history of learning problems, repeating a grade, military service, and psychiatric disorders (mainly depression). Family characteristics were also similar, including maternal and paternal age, maternal educational level, paternal occupation, number of older siblings, and family history of drug and alcohol problems. In addition, comparable proportions of mothers reported smoking cigarettes and cannabis, drinking alcoholic beverages, receiving prenatal care, and having medical or obstetrical complications when they were pregnant with the subject.

**Table 2:** Distribution of Selected Characteristics of Subjects and Parents by PCE Exposure Status

Characteristic	Prenatal and Early Childhood Exposure (*N* = 363)		Unexposed (*N* = 255)	
Year of birth	n	%	n	%
1969–1974	77	21.2%	66	25.9%
1975–1980	191	52.6%	134	52.5%
1981–1983	95	26.2%	55	21.6%
Current age (mean, sd)	40 (4)		41 (4)	
Sex at birth				
Male	134	36.9%	79	31.0%
Female	229	63.1%	176	69.0%
% White race	359	98.9%	253	99.2%
Current educational level				
High school graduate or less	18	5.0%	16	6.3%
Some college	61	16.9%	49	19.3%
Four year college grad or higher	283	78.2%	189	74.4%
Missing	1		1	
Currently employed				
Yes	325	90.0%	226	90.4%
No	36	10.0%	24	9.6%
Missing	2		5	
Current marital status				
Married or cohabitating	313	86.2%	201	78.8%
Separated, divorced, widowed,	18	5.0%	24	9.4%
Never married	32	8.8%	30	11.8%
Ever had solvent-exposed job				
Yes	54	15.3%	34	13.7%
No	299	84.7%	215	86.3%
Missing	10		6	
Ever had solvent-exposed hobby				
Yes	306	85.0%	219	86.9%
No	54	15.0%	33	13.1%
Missing	3		3	
History of learning problem				
Yes	64	17.9%	46	18.2%
No	293	82.1%	207	81.8%
Missing	6		2	
History of repeating a grade				
Yes	33	9.2%	27	10.6%
No	325	90.8%	227	89.4%
Missing	5		1	
History of military service				
Yes	21	5.8%	17	6.7%
No	342	94.2%	238	93.3%
History of mental disorder*				
Yes	152	42.3%	91	35.8%
No	207	57.7%	163	64.2%
Missing	4		1	
Mother’s age at subject’s birth (mean sd)	27 (4)		28 (4)	
Father’s age at subject’s birth (mean, sd)	30 (6)		30 (5)	
Mother’s educational level at subject’s birth				
High school graduate or less	124	34.2%	71	28.0%
Some college	101	27.8%	91	35.8%
> = Four year college graduate	138	38.0%	92	36.2%
Missing	0		1	
Father’s occupation at subject’s birth				
White collar	208	57.8%	125	49.4%
Blue collar	107	29.7%	76	30.0%
Other	45	12.5%	52	20.6%
Missing	3		2	
Mother received prenatal care during subject’s gestation				
Yes	352	99.7%	243	100.0%
No	1	0.3%	0	0.0%
Missing	10		12	
Mother smoked cigarettes during subject’s gestation				
Yes	72	23.4%	46	21.9%
No	235	76.5%	164	78.1%
Missing	56		45	
Mother consumed alcohol during subject’s gestation				
Yes	150	49.0%	91	43.3%
No	156	51.0%	119	56.7%
Missing	57		45	
Mother used cannabis during subject’s gestation				
Yes	10	3.2%	9	4.3%
No	298	96.8%	198	95.7%
Missing	55		48	
Mother had medical and obstetrical complications during subject’s gestation				
Yes	59	19.3%	52	25.1%
No	246	80.7%	155	74.9%
Missing	58		48	
Mother had occupational exposure to solvents				
Yes	45	14.8	29	14.1
No	259	85.2	177	85.9
Missing	59		49	
Birth weight (mean, sd) Missing	3428 (504) 4		3406 (556) 27	
Gestational age (mean, sd) Missing	40 (3) 13		40 (2) 13	
Multiple pregnancy	8	2.2%	10	3.9%
Subject breast fed				
Yes	198	64.9%	142	68.6%
No	107	35.1%	65	31.4%
Missing	58		48	
Number of older siblings				
0	168	46.3%	121	47.8%
1	128	35.3%	79	31.2%
2+	67	18.5%	53	20.9%
Missing	0		2	
Immediate family member was a problem drinker				
Yes	174	49.2%	103	41.9%
No	180	50.8%	143	58.1%
Missing	9		9	
Immediate family member had problems with drugs				
Yes	82	23.9%	54	22.3%
No	261	76.1%	188	77.7%
Missing	20		13	

*Includes anxiety, depression, post-traumatic stress disorder, bipolar disorder, eating disorder and schizophrenia

The Phase 2 subjects experienced a wide distribution of PCE exposure levels that encompassed several orders of magnitude (range: 0.37 to 3722 g). The distributions of PCE levels were also similar between the two phases [mean (sd): 141.6 (358.1) for Phase 1 and 150.5 (351.8) for Phase 2]. The exposure measures were based on the mass of PCE delivered to a subject’s residence through the water distribution system over the course of a year. This annual mass of PCE was diluted in approximately 90,000 gal of water, the average annual usage among Massachusetts households during their period [47]. When we converted our PCE exposure measures to annual point concentrations, we estimated that PCE concentrations in water entering the study homes ranged from less than 1 μg/L to 5197 μg/L. These levels are consistent with public water testing results during the study period [1].

Our comparison of each subject’s self-assessed exposure status to that derived from our modeled assessment revealed that only 15% of subjects considered exposed by the modeled assessment thought that their drinking water was contaminated, whereas 13% of exposed subjects thought that their water was not contaminated and 72% were unsure. Similarly, we found that 18% of subjects considered unexposed by our modeled assessment thought that their drinking water was not contaminated, while 11% thought that their drinking water was contaminated and 70% were unsure.

Overall, 23.3% of subjects reported having at least one criterion for drug use disorder over their lifetime (26.2% of exposed and 19.2% of unexposed subjects). PCE exposure during early life was associated with a modest increase in the lifetime presence of one or more diagnostic criteria for drug use disorder (adjusted Risk Ratio (RR): 1.4, 95% CI: 1.0–1.8) (Table 3). Modest to moderate associations were also generally seen for early life PCE exposure and the number of reported drug use disorder criteria --adjusted RRs were 1.3 for 2 or more criteria (95% CI:0.9–2.0), 1.6 for one criterion (95% CI: 0.9–2.8), 0.9 for 2–3 criteria (95% CI:0.5–1.9), 1.9 for 4–5 criteria (95% CI: 0.7–5.1), and 1.5 for 6 or more criteria (95% CI: 0.8–3.0, Table 3). Contrary to expectation, higher RRs were generally observed for PCE levels less than the median as compared to PCE levels greater than or equal to the median (Table 3).

**Table 3:** Lifetime Criteria of Drug Use Disorder Following Early Life Exposure to Tetrachloroethylene-contaminated Drinking Water

Outcome	Exposure Level	% Yes (n/N)	Crude	Adjusted*
			RR (95% CI)	GEE RR (95% CI)
Any (> = 1) Criteria	Any	26.2 (95/363)	1.4 (1.0,1.8)	1.4 (1.0,1.8)
	> = median	23.6 (43/182)	1.2 (0.9,1.8)	1.2 (0.8,1.7)
	> 0- < median	28.7 (52/181)	1.5 (1.1,2.1)	1.5 (1.1,2.2)
	None	19.2 (49/255)	Reference	Reference
Number of Criteria				
One Criterion	Any	12.4 (38/306)	1.6 (0.9,2.8)	1.6 (0.9,2.8)
	> = median	11.5 (18/157)	1.5 (0.8,2.8)	1.5 (0.8,2.7)
	> 0- < median	13.4 (20/149)	1.8 (1.0,3.2)	1.8 (1.0,3.4)
	None	7.6 (17/223)	Reference	Reference
Two or More Criteria	Any	17.7 (57/325)	1.3 (0.9, 1.9)	1.3 (0.9,2.0)
	> = median	15.2 (25/164)	1.1 (0.7,1.8)	1.1 (0.7,1.8)
	> 0- < median	19.9 (32/161)	1.5 (0.9,2.3)	1.5 (0.9,2.4)
	None	13.4 (32/238)	Reference	Reference
Two to Three Criteria	Any	6.9 (20/288)	1.2 (0.6,2.3)	0.9 (0.5,1.9)
	> = median	7.3 (11/150)	1.2 (0.6,2.7)	0.9 (0.4,2.0)
	> 0- < median	6.5 (9/138)	1.1 (0.5,2.5)	1.0 (0.4,2.2)
	None	5.9 (13/219)	Reference	Reference
Four to Five Criteria	Any	4.3 (12/280)	1.5 (0.6,4.0)	1.9 (0.7,5.1)
	> = median	2.8 (4/143)	1.0 (0.3,3.4)	1.4 (0.4,4.6)
	> 0- < median	5.8 (8/137)	2.1 (0.7,5.8)	2.3 (0.8,6.9)
	None	2.8 (6/212)	Reference	Reference
Six or More Criteria	Any	8.5 (25/293)	1.4 (0.8,2.7)	1.5 (0.8,3.0)
	> = median	6.7 (10/149)	1.1 (0.5,2.5)	1.2 (0.5,2.8)
	> 0- < median	10.4 (15/144)	1.8 (0.9,3.6)	1.9 (0.9,4.0)
	None	5.9 (13/219)	Reference	Reference

*Adjusted for subject’s age, father’s occupation, mother’s educational level

When these data were stratified by sex, we found that early life PCE exposure remained associated with a modest increase in the lifetime presence of one or more diagnostic criteria for drug use disorder among females (adjusted RR: 1.5, 95% CI: 1.0–2.3) but not males (adjusted RR: 1.0, 95% CI:0.6–1.5). The *p* value for the sex interaction term was 0.10 which reflects, in part, the low proportion of male respondents in the study (Table 2).

Compared to unexposed subjects, PCE exposed subjects were more likely to report having most of the individual criteria for drug use disorder. For example, they were more likely to report neglecting major roles due to drug use (5.8 vs. 3.1%), physical and psychological problems related to drug use (8.3 vs. 4.3%), and giving up activities due to drug use (3.9 vs. 2.0%) (Table 4). We also found that all types of drug use were more common among subjects with one or more criteria for drug use disorder. Compared to subjects without any criteria for drug use disorder, those with at least one criterion were more likely to use/misuse multiple drugs (73.5% vs. 28.4%), including cannabis (93.2% vs. 70.2%), crack/cocaine (52.8% vs. 12.6%), hallucinogens (60.9% vs. 21.7%), inhalants (28.4% vs. 6.6%), methamphetamine (11.9% vs. 1.1%), heroin (11.9% vs. 0.4%), prescription pain relievers (42.0% vs. 5.8%), prescription tranquilizers (24.7% vs. 3.8%), and prescription stimulants (30.9% vs. 4.3%).

**Table 4:** Distribution (%) of Drug Use Criteria Overall and According to Early Life Exposure to PCE Exposure

	Overall *N* = 618		Exposed N = 363		Unexposed N = 255	
	n	%	n	%	n	%
Drug Use Criteria						
Hazardous use	66	10.7	43	11.9	23	9.0
Social/interpersonal problems related to use	29	4.7	20	5.5	9	3.5
Neglected major roles to use	29	4.7	21	5.8	8	3.1
Experienced withdrawal	50	8.1	29	8.0	21	8.2
Developed tolerance	54	8.7	34	9.4	20	7.8
Used larger amounts/longer	50	8.1	35	9.6	15	5.9
Repeated unsuccessful attempts to quit or control use	71	11.5	47	13.0	24	9.4
Much time spent using	59	9.6	38	10.5	21	8.2
Physical/psychological problems related to use	41	6.6	30	8.3	11	4.3
Activities given up to use	19	3.1	14	3.9	5	2.0
Developed craving	64	10.4	45	12.4	19	7.5

## Discussion

These results suggest that there is a modest increase in the risk of meeting one or more criteria for drug use disorder among adults with any exposure to PCE-contaminated drinking water during gestation and early childhood. No dose-response relationships were observed with increasing levels of PCE exposure. Nevertheless, the present results are consistent with our previous finding that illicit drug use is increased among individuals with early life exposure to PCE-contaminated drinking water [27]. In that study, specific increases in risk were seen for using crack/cocaine, psychedelics/hallucinogens, club/designer drugs and heroin. Because the use of these drugs may lead to further uncontrollable use [28, 29], it is plausible that early life PCE exposure also increases the risk of having one or more criteria for drug use disorder.

These findings should be interpreted in light of the study limitations. The first is likely exposure misclassification. Because historical PCE exposure measurements were impossible to obtain from subject residences, we estimated PCE exposure using water distribution modeling software that was modified to incorporate a leaching and transport model [41, 48]. The model was applied to water distribution system conditions in 1980 and was assumed to be representative of the entire exposure period. This assumption was supported by observation that the distribution systems changed little between the late 1960s and the 1980s, except for adding water sources to accommodate population growth. Furthermore, the exposure assessment predicted the annual mass of PCE delivered to each subject’s residence during gestation and early childhood, and did not incorporate information on water consumption and bathing habits due to poor recall of these behaviors. While results from validation studies indicate reasonable correlation between our exposure estimates and PCE concentrations in historical water samples [49, 50], non-differential exposure misclassification likely biased the findings from dichotomous comparisons (e.g., any exposure vs. none) towards the null [50]. The expected direction of bias for comparisons involving three exposure levels (e.g., high, low, unexposed) is more difficult to predict. While it is possible that the lack of a dose-response relationship according to PCE levels may reflect bidirectional misclassification between the low and high exposure levels, it is also possible that a non-linear dose-response relationship is present as has been observed for PCE and many other endocrine disrupting chemicals [51].

Another limitation stems from the use of self-reports as the source of information on the criteria for drug use disorder and did not capture diagnoses made by clinicians who were trained in addiction medicine. While the prevalence of drug use among study subjects was similar or higher than reported in independent surveys of Cape Cod and other Massachusetts residents [52], some underreporting of the criteria was likely in this questionnaire format. However, since most subjects did not know their exposure status, underreporting was likely to be non-differential and so would not have affected the observed risk ratios [50].

Still another limitation stems from possible residual confounding because of missing data on risk factors for drug use disorder (such as family dynamics and peer pressure). However, in order to account for the associations observed in this study, these factors would need to be causally related to PCE exposure, an unlikely scenario given the irregular pattern of the PCE contamination across the neighborhoods of Cape Cod. In fact, our prior analyses of this cohort also found little or no confounding for the outcomes being investigated [27, 53].

A further limitation stems from the low response rate. Although this problem reduced the statistical power of the study, the following evidence suggests that it did not result in selection bias. First, many available characteristics of Phase 1 and 2 participants and non-participants were similar, including PCE exposure status. Second, while Phase 2 participants were more likely to be female, and have college-educated mothers, these differences were equally present for exposed and unexposed non-participants. Third, losses stemming from the death of potential subjects (*N* = 117) were small and unrelated to initial PCE exposure status. Our review of death records from the Massachusetts Registry of Vital Records and Statistics and the National Death Index suggested that seven of the 117 deaths were associated with substance use; four of these deaths occurred among exposed subjects and three occurred among unexposed subjects.

Both animal and human studies have found neurotoxic effects following PCE exposure [6]. Because of its small size and lipid solubility, PCE easily crosses the blood brain barrier and selectively concentrates in the brain and other lipophilic tissue. Epidemiologic studies of adults with occupational exposure to PCE and related solvents have reported increases in anxiety and depression [8, 9, 11, 16–18] and impairments in cognition, memory, attention, executive function and trigeminal nerve and vestibular function [8–15].

Studies of the neurotoxic effects among individuals with early life exposure to organic solvents have produced mixed results. Eskenazi et al. found no deficits in intellectual ability, motor skills or memory among pre-school children whose mothers had jobs involving solvent exposure during pregnancy [19]. In addition, no meaningful differences were seen in two studies of cognitive and abnormal behavioral function among children attending a nursery school and day care center who were exposed to PCE from nearby dry cleaning facilities [20, 21]. Our prior cohort study on the reproductive and developmental effects of prenatal and early postnatal PCE exposure also did not observe any associations with disorders of attention, learning, and behavioral control throughout childhood [54]. In contrast, Till et al. found that pre-school children whose mothers were exposed to organic solvents during pregnancy had lower scores on language tests, reduced graphomotor skills, and more behavioral problems than unexposed children [22]. In addition, Laslo-Baker et al. found that preschool children with prenatal exposure to organic solvent mixtures scored lower on tests of general intelligence, language and motor skills [23]. Pele et al. also found a greater frequency of behaviors indicative of attention deficits, hyperactivity, and aggression [24] and Costet et al. found increased externalizing behavior among children with prenatal exposure to solvents [25]. These disparate findings may stem from varying exposure levels and the use of different measures of neurobehavioral measures as outcomes.

An important feature of these early life studies is their focus on short-term effects in young children. To the best of our knowledge, only three prior studies --ours and two others-- have investigated the long-term neurotoxic impacts of early life exposure to solvents. An Israeli study found a 3.4-fold increased risk of schizophrenia among offspring of parents who worked in dry cleaning during their gestation and childhood [55], and a Danish study found a 3.2-fold increased risk of schizophrenia among individuals who were exposed to high levels of benzene air pollution during early life [56].

Our cohort study investigated a wider variety of neurotoxic effects, including drug use among individuals with prenatal and early childhood PCE exposure [26, 27, 57]. Moreover, our prior analyses found that individuals who were exposed to PCE-contaminated drinking water in early life experienced 30–40% increases in the risk of using multiple drugs as a teenager or as an adult [27]. We also found that early life PCE exposure was associated with increased risks of mood disturbances and diminished performance on tests of learning, memory, attention, and executive functioning [26].

The mechanism by which PCE and related solvents may cause neurotoxic effects is presently unknown [6]. Furthermore, to the best of our knowledge, no one has investigated how early life PCE exposure might contribute to the multifactorial etiology of drug use disorder [30, 31]. However, available evidence suggests that PCE may exert neurotoxic actions via the peroxidation of cell membrane lipids [32], changes in the fatty acids in the brain [33], demyelination of nerve cells [34], and changes in ligand-gated ion channel activity involving the following receptors: GABA_A_, glycine, NMDA, glutamate kainite, and AMPA (e.g., (35–39)). It has been postulated that exposure to agents such as ethanol during synaptogenesis can trigger substantial apoptotic neurodegeneration because these agents interfere with the action of neurotransmitters and GABA_A_ receptors [58].

The mechanism by which the neurotoxic impact of PCE exposure may be sex dependent is also not well understood. However, sex differences are quite plausible via differences in toxicokinetics. In this instance, possible explanations for a greater impact of early life exposure to chlorinated solvents such as PCE among females include increased dermal absorption [59], preferential sequestration in fat tissue which is found at higher levels in females [60], and permeability differences in the blood brain barrier [59].

In summary, the results of this study suggest that the criteria associated with drug use disorder are modestly increased among adults exposed to PCE-contaminated drinking water during gestation and early childhood. Because this study has several limitations, these findings should be confirmed in follow-up investigations of other exposed populations with more diverse racial and socioeconomic characteristics. Because PCE remains a common contaminant of public drinking water supplies, it is important to determine its long-term impacts on behavioral health.

## Conclusions

We found a modest increase in the risk of having criteria for drug use disorder among adults who were exposed to PCE-contaminated drinking water during gestation and early childhood. Because this study has several limitations and PCE is a common environmental contaminant, additional long-term studies of diverse populations are needed to determine its possible impact on behavioral health.

## Data Availability

The datasets generated and analyzed during this study are not publically available due to IRB restrictions. Non-identifiable data are however available from the authors upon reasonable request and with permission from the IRBs at Boston University and the Massachusetts Department of Public Health.

## Abbreviations

AC: Asbestos Cement
AMPA: Alpha-amino-3-hydroxy-5-methyl-4-isoxazolepropionic acid
AUDADIS: Alcohol Use Disorder and Associated Disabilities Interview Schedule
CI: Confidence Interval
DAG: Directed Acyclic Graph
DSM5: Diagnostic and Statistical Manual of Mental Disorders, Fifth Edition
EPA: US Environmental Protection Agency
EPANET: US EPA Water Modeling Softwar
GABAA: Gamma-aminobutyric Acid (a)
GEE: Generalized Estimating Equations
NMDA: N-methyl-D-aspartate
PCE: Tetrachloroethylene
RR: Risk Ratio
SD: Standard Deviation
VL: Vinyl-Lined
